# Sex Differences in Neoplastic Progression in Barrett’s Esophagus: A Multicenter Prospective Cohort Study

**DOI:** 10.3390/cancers14133240

**Published:** 2022-07-01

**Authors:** Carlijn A. M. Roumans, Pauline A. Zellenrath, Ewout W. Steyerberg, Iris Lansdorp-Vogelaar, Michael Doukas, Katharina Biermann, Joyce Alderliesten, Gert van Ingen, Wouter B. Nagengast, Arend Karrenbeld, Frank ter Borg, Mariska Hage, Pieter C. J. ter Borg, Michael A. den Bakker, Alaa Alkhalaf, Frank C. P. Moll, Lieke Brouwer-Hol, Joop van Baarlen, Rutger Quispel, Arjan van Tilburg, Jordy P. W. Burger, Antonie J. P. van Tilburg, Ariadne H. A. G. Ooms, Thjon J. Tang, Mariëlle J. L. Romberg-Camps, Danny Goudkade, Marco J. Bruno, Dimitris Rizopoulos, Manon C. W. Spaander

**Affiliations:** 1 Department of Gastroenterology & Hepatology, Erasmus MC University Medical Center, 3015 GD Rotterdam, The Netherlands; c.roumans@erasmusmc.nl (C.A.M.R.); p.zellenrath@erasmusmc.nl (P.A.Z.); m.bruno@erasmusmc.nl (M.J.B.); 2 Department of Public Health, Erasmus MC University Medical Center, 3015 GD Rotterdam, The Netherlands; e.steyerberg@erasmusmc.nl (E.W.S.); i.vogelaar@erasmusmc.nl (I.L.-V.); 3 Department of Biomedical Data Sciences, Leiden University Medical Center, 2333 ZA Leiden, The Netherlands; 4 Department of Pathology, Erasmus MC University Medical Center, 3015 GD Rotterdam, The Netherlands; m.doukas@erasmusmc.nl (M.D.); k.biermann@erasmusmc.nl (K.B.); a.ooms@pathan.nl (A.H.A.G.O.); 5 Department of Gastroenterology & Hepatology, Albert Schweitzer Ziekenhuis, 3318 AT Dordrecht, The Netherlands; jalderliesten@asz.nl; 6 Laboratorium voor Pathologie, 3318 AL Dordrecht, The Netherlands; gingen@paldordrecht.nl; 7 Department of Gastroenterology & Hepatology, Groningen University Medical Center, 9713 GZ Groningen, The Netherlands; w.b.nagengast@umcg.nl; 8 Department of Pathology, Groningen University Medical Center, 9713 GZ Groningen, The Netherlands; a.karrenbeld@path.azg.nl; 9 Department of Gastroenterology & Hepatology, Deventer Ziekenhuis, 7416 SE Deventer, The Netherlands; f.terborg@dz.nl; 10 Department of Pathology, Deventer Ziekenhuis, 7416 SE Deventer, The Netherlands; m.hage@dz.nl; 11 Department of Gastroenterology & Hepatology, Ikazia Ziekenhuis, 3083 AN Rotterdam, The Netherlands; pcj.ter.borg@ikazia.nl; 12 Department of Pathology, Ikazia Ziekenhuis, 3083 AN Rotterdam, The Netherlands; bakkerma@maasstadziekenhuis.nl; 13 Department of Pathology, Maasstad Ziekenhuis, 3079 DZ Rotterdam, The Netherlands; 14 Department of Gastroenterology & Hepatology, Isala Klinieken Zwolle, 8025 AB Zwolle, The Netherlands; a.alkhalaf@isala.nl; 15 Department of Pathology, Isala Klinieken Zwolle, 8025 AB Zwolle, The Netherlands; f.c.p.moll@isala.nl; 16 Department of Gastroenterology & Hepatology, Maasstad Ziekenhuis, 3079 DZ Rotterdam, The Netherlands; holl@maasstadziekenhuis.nl; 17 Department of Pathology, Medisch Spectrum Twente, 7512 KZ Enschede, The Netherlands; j.vanbaarlen@laborpath.nl; 18 Laboratorium Pathologie Oost-Nederland, 7555 BB Hengelo, The Netherlands; 19 Department of Gastroenterology & Hepatology, Reinier de Graaf Gasthuis, 2625 AD Delft, The Netherlands; r.quispel@rdgg.nl; 20 Department of Pathology, Reinier de Graaf Gasthuis, 2625 AD Delft, The Netherlands; a.vantilburg@rdgg.nl; 21 Department of Gastroenterology & Hepatology, Rijnstate Ziekenhuis, 6815 AD Arnhem, The Netherlands; jburger@rijnstate.nl; 22 Department of Gastroenterology & Hepatology, Franciscus Gasthuis & Vlietland, 3045 PM Rotterdam, The Netherlands; a.vantilburg@franciscus.nl; 23 Pathan, Pathologisch Laboratorium, 3045 PM Rotterdam, The Netherlands; 24 Department of Gastroenterology & Hepatology, IJsselland Ziekenhuis, 2906 ZC Capelle a/d Ijssel, The Netherlands; tjtang@ysl.nl; 25 Department of Gastroenterology & Hepatology, Zuyderland Medisch Centrum, 6162 BG Sittard, The Netherlands; m.camps@zuyderland.nl; 26 Department of Pathology, Zuyderland Medisch Centrum, 6162 BG Sittard, The Netherlands; d.goudkade@zuyderland.nl; 27 Department of Biostatistics, Erasmus University Medical Center, 3015 GD Rotterdam, The Netherlands; d.rizopoulos@erasmusmc.nl

**Keywords:** Barrett’s esophagus, sex, acceleration rate, neoplastic progression

## Abstract

**Simple Summary:**

Barrett’s esophagus (BE) is the only known precursor lesion of esophageal adenocarcinoma (EAC). Endoscopic surveillance plays an important role in the timely detection of neoplastic progression. However, the cost-effectiveness of current surveillance strategies is debatable. Previous studies have shown that male Barrett’s patients have lower neoplastic progression risk than females. However, these studies do not provide a more practical translation of these sex disparities into different surveillance intervals. The current multicenter prospective cohort study aimed to evaluate sex differences in 868 BE patients; not only with respect to neoplastic progression risk, but also concerning the difference in time to detection of high-grade dysplasia (HGD)/EAC: time to neoplastic progression was estimated to be almost twice as low in males than in females. In contrast, the stage of neoplasia appeared to be higher in females. Our results can guide future discussions for sex-specific guidelines, supporting the implementation of neoplastic risk stratification per individual patient in BE surveillance.

**Abstract:**

Recommendations in Barrett’s esophagus (BE) guidelines are mainly based on male patients. We aimed to evaluate sex differences in BE patients in (1) probability of and (2) time to neoplastic progression, and (3) differences in the stage distribution of neoplasia. We conducted a multicenter prospective cohort study including 868 BE patients. Cox regression modeling and accelerated failure time modeling were used to estimate the sex differences. Neoplastic progression was defined as high-grade dysplasia (HGD) and/or esophageal adenocarcinoma (EAC). Among the 639 (74%) males and 229 females that were included (median follow-up 7.1 years), 61 (7.0%) developed HGD/EAC. Neoplastic progression risk was estimated to be twice as high among males (HR 2.26, 95% CI 1.11–4.62) than females. The risk of HGD was found to be higher in males (HR 3.76, 95% CI 1.33–10.6). Time to HGD/EAC (AR 0.52, 95% CI 0.29–0.95) and HGD (AR 0.40, 95% CI 0.19–0.86) was shorter in males. Females had proportionally more EAC than HGD and tended to have higher stages of neoplasia at diagnosis. In conclusion, both the risk of and time to neoplastic progression were higher in males. However, females were proportionally more often diagnosed with (advanced) EAC. We should strive for improved neoplastic risk stratification per individual BE patient, incorporating sex disparities into new prediction models.

## 1. Introduction

Barrett’s esophagus (BE) is a precancerous condition related to gastroesophageal reflux disease (GERD); chronic inflammation of esophageal mucosa results in the replacement of squamous epithelium by metaplastic intestinal epithelium. This predisposes the development of low-grade dysplasia (LGD), high-grade dysplasia (HGD) and eventually esophageal adenocarcinoma (EAC) [1,2]. In recent decades, the incidence of EAC in the Western population has increased rapidly and continues to rise [3,4]. With an estimated 5-year survival rate of <20%, EAC is considered one of the deadliest cancers of the gastrointestinal tract [5]. BE patients undergo endoscopic surveillance in order to detect neoplasia at an early stage, which enables endoscopic curable resection with a considerable higher survival rate than more advanced EAC.

Within the course from GERD to BE and eventually to HGD/EAC, the proportion of affected males compared to females increases. For instance, the prevalence of BE is almost three times higher in males than in females [6]. Consequently, males represent the majority of the BE population. This sex difference is also reflected in BE study cohorts. Conclusions with respect to neoplastic progression may therefore be particularly applicable to males. Studies addressing the difference in neoplastic progression between males and females show that male sex is an independent risk factor for progression from BE to HGD/EAC [4,7,8,9]. A potential explanation may be the onset of BE at an older age in females than in males due to menopause-related hormonal differences [7,10,11].

These studies have clearly revealed the vital importance of research into a different approach between sexes with respect to surveillance intervals, as for now, current practices do not anticipate accordingly. Intervals between two subsequent endoscopies are the same for males and females. Given the lower neoplastic progression risk for females, they might be subjected to a more stringent surveillance protocol than necessary. However, current studies do not provide a more practical translation of the sex disparities with respect to the neoplastic progression risk into the differences in surveillance interval.

Therefore, it would be valuable to assess the differences in time to neoplastic progression between males and females. In other words: do males progress faster to HGD/EAC compared to females? In summary, this study aimed to evaluate the difference between sexes in (1) probability of and (2) time to neoplasia, as well as (3) sex differences in the stage distribution of neoplasia in BE patients.

## 2. Materials and Methods

### 2.1. Study Design

We conducted this study within a large multicenter prospective cohort of 868 patients with BE, which has been described before [12]. All patients were included between November 2003 and February 2020 from 3 university medical centers and 15 regional hospitals throughout the Netherlands. All patients underwent endoscopic surveillance according to the guidelines of the American College of Gastroenterology [13]. 

**Figure 1:**
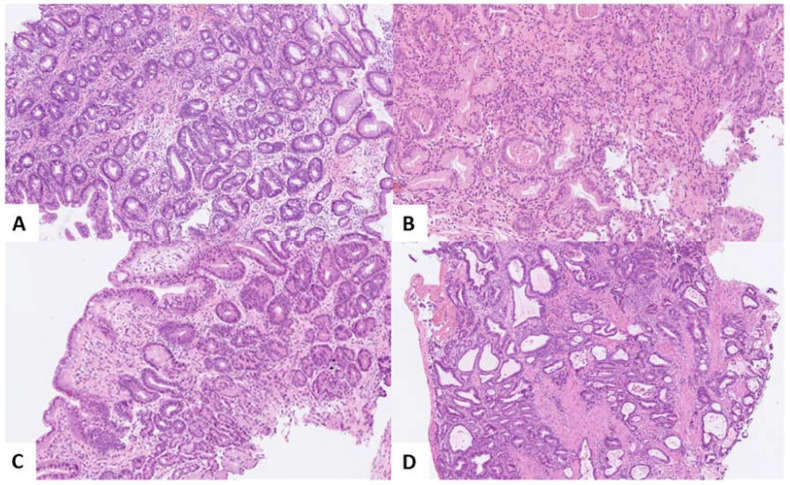
Hematoxylin–eosin staining of (**A**) Barrett’s esophagus (BE) without dysplasia (NDBE); (**B**) BE with low-grade dysplasia (LGD); (**C**) BE with high-grade dysplasia (HGD); and (**D**) esophageal adenocarcinoma (EAC). Original magnifications ×50 [A and D], ×80 [C] and ×100 [B]).

At index endoscopy, demographic information was collected. Before every follow-up (FU) endoscopic procedure, patients were asked to fill out a short questionnaire concerning symptoms of GERD, smoking, alcohol consumption, use of medication, height, and weight. Endoscopic procedures were performed according to a standardized protocol. Before taking biopsies, endoscopic landmarks, such as the diaphragm impression, gastroesophageal junction, and squamocolumnar junction, were reported. The presence of esophagitis was classified according to the Los Angeles Classification and abnormalities were noted, including nodules, ulcers, and erosions. Targeted biopsies were taken from mucosal abnormalities if present. Furthermore, quadrant biopsies were taken from every 2 cm of the Barrett segment according to the Seattle protocol. Biopsy slides were graded by an experienced local pathologist to assess the presence of BE and to define the grade of dysplasia. If discordant, an expert panel of pathologists reviewed the slides to define the final conclusion. Based on the fact that the histopathological assessment of either reactive changes, no dysplasia, and any grade of dysplasia in BE surveillance can be challenging [14], the need for consensus agreement has been incorporated in the guidelines [15]. This study was performed according to the Dutch guidelines. Histopathology could be graded as no dysplasia (ND), indefinite for dysplasia (IND), low-grade dysplasia (LGD), high-grade dysplasia (HGD) or esophageal adenocarcinoma (EAC) (Figure 1). Both HGD and EAC were labelled as neoplastic progression and were staged according to the 8th edition of the TNM staging of the American Joint Committee on Cancer [16]. Patients without dysplasia underwent upper endoscopy with biopsy sampling every 3 years. Patients with IND, LGD or a BE segment of >10 cm underwent surveillance every year. The endpoint of the study was the detection of HGD or EAC.

### 2.2. Study Population

To be included, patients had to have known or newly diagnosed BE of at least 2 cm in length according to the Prague C&M criteria [17], histologically confirmed by the presence of intestinal metaplasia. Patients with a history of HGD or esophageal malignancy were excluded. Patients who had an interval of less than 6 months between the index endoscopy and diagnosis of HGD/EAC were excluded from this study to prevent the inclusion of prevalent cases.

### 2.3. Ethics

The study protocol was approved by the institutional review board of Erasmus MC University Medical Center. Written informed consent was obtained from all patients prior to inclusion.

### 2.4. Statistical Analysis

In this analysis, we defined HGD or EAC as the development of neoplastic progression. The risk of the detection of the event for both sexes was estimated using a Cox proportional hazard model, adjusted for time-varying covariates (length of BE and esophagitis) and a baseline covariate (age). The time to neoplastic progression to the event for both sexes was estimated using the same covariates in an accelerated failure time model. A sensitivity analysis was performed to test the results for robustness, in which the time-varying covariate esophagitis was substituted for the baseline LGD. The length of BE (≤2 cm vs. 3 cm and more), esophagitis (present vs. absent), sex (male vs. female), and baseline LGD (NDBE vs. LGD) were used as a binary variable; age was used as a continuous variable. Due to the limited number of patients developing the event for the stage distribution, descriptive statistics were used. The statistical difference of potential differences in baseline characteristics was tested using a chi-square test for binary variables and using a Mann–Whitney U test for continuous variables. *p*-values < 0.05 were considered statistically significant. Analysis was performed with R [18], version 3.4.1.

## 3. Results

### 3.1. Baseline Characteristics

A total of 868 BE patients were included, consisting of 639 (74%) males and 229 females (Table 1). Males were followed for a median duration of 7.2 years (IQR 3.2–12.3) and females for a median duration of 7.1 years (3.7–12.0). During this period, a median of 4 (IQR 2–6) endoscopies were performed in both sexes. The mean age at diagnosis was 60 years (IQR 52–69) in males, and 65 years (IQR 57–71) in females. GERD was present in 31% and the median length of the BE segment was 4 cm (IQR 2.0–6.0 in males, IQR 3.0–6.0 in females) in both sexes. Esophagitis was present in 62 (10%) males and 20 (9%) females. The male cohort carried a higher burden of comorbidity, including tobacco use and alcohol intake. The female cohort had a significantly higher age at diagnosis, higher BMI, and more PPI use. There were no other significant differences between males and females. 

### 3.2. Risk of Neoplastic Progression

Out of all patients included, 61 (7%) developed HGD/EAC during surveillance. Of the 52 (8%) males with neoplastic progression, 37 (71%) were diagnosed with HGD and 15 (29%) with EAC. This proportion was different within females. Of the nine (4%) females with neoplastic progression, four (44%) had HGD and five (56%) had EAC (Table 2). Although females more often had EAC than HGD compared to males, the risk of neoplastic progression was estimated to be more than twice as high among males than females (HR 2.3, 95% CI 1.1; 4.6) (Table 3). If only the risk of HGD was taken into consideration, the difference was even larger (HR 3.8, 95% CI 1.3–10.6). In the sensitivity analyses, in which we adjusted for baseline LGD instead of the presence of esophagitis, the results were robust for the risk of progression to HGD (HR 3.2, 95% CI 1.1–9.0). However, the difference in the overall neoplastic progression risk was not statistically significant (HR 1.9, 95% CI 0.9–3.9) (Supplementary Table S1).

### 3.3. Time to Neoplastic Progression

The median time to neoplastic progression was 4.2 years (IQR 2.0; 6.3); this was particularly shorter for males than for females (2.1 years (IQR 1.0; 4.3) versus 4.6 years (IQR 2.0; 6.4), respectively). The average time to develop HGD/EAC was approximately 60% shorter for males than for females (AR 0.40, 95% CI 0.19–0.86) and almost twice as fast if only HGD would be taken into consideration (AR 0.52, 95% CI 0.29–0.95) (Table 3). For HGD as a study endpoint, the results were robust in a sensitivity analysis, in which they were adjusted for baseline LGD instead of the presence of esophagitis (AR 0.46, 95% CI 0.22–0.96). However, for the combined endpoint of HGD/EAC, the average time to develop neoplastic progression was still longer in females than males, and the results were not statistically significant (AR 0.52, 95% CI 0.34–1.10) (Supplementary Table S1).

### 3.4. Stage Distribution of Neoplastic Progression

**Figure 2:**
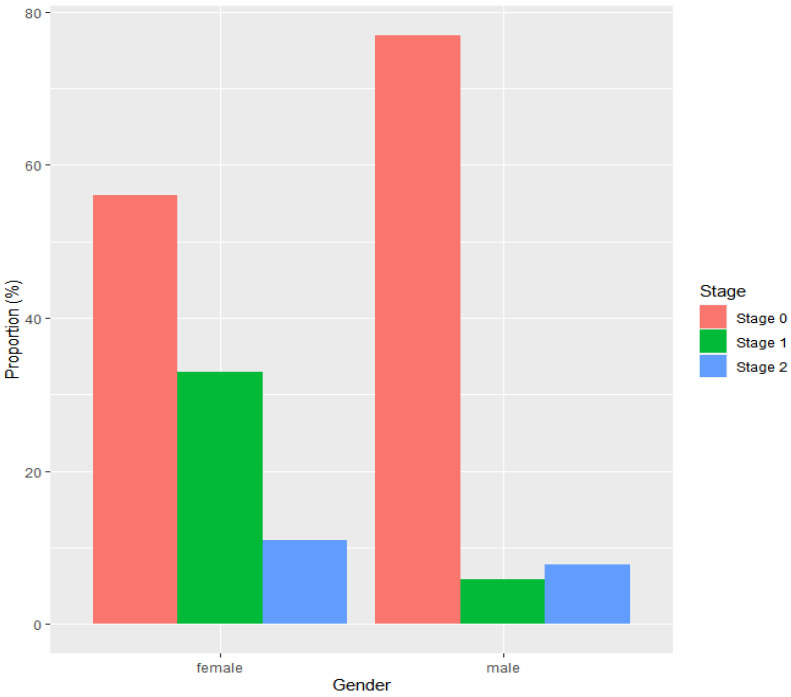
Stage distribution of neoplasia according to sex, with stage 0 including TisN0M0 and HGD, stage 1 including T1N0M0 and T2N0M0, and stage 2 including T3N0M0 and T1/T2N1M0. Data for 5 male patients were missing.

Neoplastic progression was detected in 52 males, among which 40 (77%) were diagnosed with stage 0 (HGD or pTisN0M0), 4 (8%) were diagnosed with stage 1 (pT1N0M0 or pT2N0M0 EAC), and 3 (6%) were diagnosed with stage 2 (pT3N0M0 or pT1/T2N1M0 EAC). For 5 (10%) males with neoplasia, it was not possible to retrieve information with respect to the stage of neoplastic progression at diagnosis. The stage distribution was different in females with neoplastic progression. A total of nine females had developed neoplastic progression; 5 (56%) were diagnosed with stage 0, 3 (33%) with stage 1, and 1 (11%) with stage 2 (Figure 2).

## 4. Discussion

In this large multicenter prospective cohort study, we evaluated the differences in patients with BE in risk of and time to neoplastic progression between sexes. Males were more likely to develop neoplasia, with an overall neoplastic progression risk that was twice as high compared to females. In particular, the risk of developing HGD was almost four times higher among males. Furthermore, the time to neoplastic progression was significantly shorter in males, who developed neoplasia on average twice as fast as females. On the other hand, female patients who progressed to neoplasia had EAC proportionally more often than HGD and tended to have a higher stage of neoplasia compared to the male population in our study. 

Our findings with respect to a higher absolute risk of neoplastic progression and shorter time to neoplasia in males than in females are in line with previous results [9,19,20,21]. These findings suggest that females might benefit less from current surveillance intervals than males. Prolonging surveillance intervals for patients with a low neoplastic progression risk could increase the cost-effectiveness of BE surveillance substantially, which is worth considering given the debatable cost-effectiveness of current surveillance strategies [21,22,23]. However, while the number (and thus costs) of surveillance endoscopies will decrease with prolonging surveillance intervals, the risk of interval carcinoma’s will simultaneously increase. The incidence of interval carcinoma’s must stay within acceptable ranges in order to safely alter surveillance strategies. Unfortunately, practical implications of known sex disparities into safe surveillance intervals are lacking. As a result, sex is not yet considered an independent variable to determine surveillance intervals for individual BE patients. The present study provides some valuable new insights that could guide future discussions about making sex-specific BE surveillance guidelines.

To our knowledge, this is the first study indicating the difference in average time to neoplastic progression between both male and female BE patients. The use of such a study design is particularly appealing if a disease is the result of a process with a known sequence of intermediary stages, as is the case in BE [24]. The malignant potential of BE starts with genetic and epigenetic alterations that result in the dysfunction of tumor suppressor genes or proto-oncogenes, such as p16, p53 and Hox genes [25]. These (epi)genetic changes influence the cell’s ability to control the cell cycle, causing affected Barrett cells to be more prone to the development of LGD, HGD and eventually EAC. Our results show that males progress twice as fast through the carcinogenic cascade. This might indicate that all of the pre-stages progress twice as fast, and that the overall expected time until progression to EAC is half the time in males compared to females. These results support the implementation of longer surveillance intervals for females. However, surveillance intervals cannot automatically become twice as long, since more parameters than just sex must be taken into account while defining optimal surveillance intervals. Recently, sex has been included as one of the multiple parameters in a promising prediction model to stratify neoplastic progression risk per individual BE patient [26]. In this model, the longitudinal evolution of the grade of dysplasia and several immunohistochemical biomarkers along endoscopic and demographic (including sex) parameters were included. The design of this model allows to update the neoplastic progression risk of an individual BE patient at every surveillance endoscopy. This model showed good results in adjusting current surveillance intervals while retaining effectiveness in the timely detection of neoplastic progression [27]. 

In addition to the higher neoplastic progression risk and shorter time to HGD/EAC in males, our study also showed that females progressing to neoplasia tended to have a higher stage at diagnosis in comparison to males. This is worrisome, since this can seriously reduce treatment options and possibly survival, as treatment at an early stage enables an endoscopic curable resection with a considerable higher survival rate than the treatment of more advanced EAC. Additionally, it implicates that with prolonging surveillance intervals for females, the risk of (advanced) interval carcinomas may increase. These unfavorable outcomes discourage the implementation of prolonged surveillance intervals for females. However, when evaluating these observed sex disparities in the stage of dysplasia, it has to be considered that only nine females progressed to HGD/EAC in our cohort. This number is too small to draw firm conclusions and, therefore, studies with a larger number of female progressors are needed to validate our findings.

Overall, our results support the idea that the course from BE to EAC might be different in males than in females. Several explanations can be proposed. First, recent studies have provided evidence that the androgen/estrogen balance may be a factor related to the development of EA in BE patients, in which higher levels of androgens relative to estrogens may favor cellular proliferation and tumor growth [28,29]. This would explain why males seem to develop neoplasia particularly faster compared to young/premenopausal females (with high levels of estrogens), but similarly to older/postmenopausal females (with lower levels of estrogens). This suggests that females might enter the carcinogenic cascade later in life, probably due to menopause, but show neoplastic patterns to those of BE males afterwards. Based on these results, the serum levels of androgen/estrogen might even be more relevant for neoplastic risk prediction than sex alone. More research is needed to further elucidate the hypothesis that sex steroid hormones may explain sex differences within the EAC pathogenesis in BE patients. Second, our cohort showed a number of differences in baseline characteristics. For example, smoking was more common in males, which is associated with an increased risk of neoplastic progression [30]. To retain power of our statistical models, it was not possible to adjust for all variables.

Our study has several strengths. Patients were followed prospectively according to a stringent scheme, during a long follow-up period. In addition, a standardized endoscopy and biopsy protocol was used. Furthermore, biopsy slides were assessed by an experienced pathologist to assess the grade of dysplasia. However, several limitations of this study should also be acknowledged. Inherent to the prospective study design, we cannot exclude that some patients will develop HGD or EAC in the future. This may bias our findings of sex being an independent predictor for neoplastic progression. Furthermore, HGD or EAC were detected in only a small number of females despite working with a large prospective BE cohort due to the low neoplastic progression rate in BE patients. 

## 5. Conclusions

In conclusion, our study shows that both the risk of and time to neoplastic progression is higher in BE males than in females. Furthermore, this is the first study to demonstrate that males accelerate faster trough the different stages of dysplasia than females. These findings support that sex-specific guideline recommendations are important to strive for, in which females might benefit from longer surveillance intervals than males. However, the stage of neoplasia seems to be higher in females compared to males. This observation must be taken seriously, as it suggests that prolonging surveillance intervals for females might not be safe. Future research should focus on the differential aspects of neoplastic progression in BE between males and females; for example, by using multivariable prediction models to estimate an individualized neoplastic progression risk which can be updated over time. In such predictions, it might be valuable to incorporate the potential hormonal influences on sex disparities within the BE population.

## Figures and Tables

**Table 1:** Baseline characteristics.

Variables		Males (*n* = 639)	Females (*n* = 229)	*p*-Value
FU time (median, IQR)		7.2 years (3.2–12.3)	7.1 years (3.7–12.0)	0.91
n° of FU (median, IQR)		4.0 (2.0–6.0)	4.0 (2.0–6.0)	0.93
Age (median, IQR)		60 years (52–69)	65 years (57–71)	**<0.001**
GERD (%)		196 (31%)	70 (31%)	0.94
PPI use (%)		526 (82%)	208 (91%)	**0.03**
NSAID use (%)		28 (4%)	10 (4%)	1.00
Smoking (%)	current	140 (23%)	36 (16%)	**<0.001**
	former	327 (51%)	78 (34%)	
	never	166 (23%)	108 (47%)	
Alcohol (%)	current	531 (83%)	129 (56%)	**<0.001**
	ever	59 (9.2%)	26 (11%)	
	never	44 (6.9%)	66 (29%)	
BMI (median, IQR)		26.5 kg/m^2^ (24.7–29.1)	27.3 kg/m^2^ (24.8–31.4)	**0.001**
Length of BE in cm (median, IQR)		4.0 (2.0–6.0)	4.0 (3.0–6.0)	0.97
Esophagitis present (%)		62 (9.7%)	20 (8.7%)	0.81

BMI = body mass index; BE = Barrett’s esophagus; FU = follow up; GERD = gastro-esophageal reflux disease; HGD = high-grade dysplasia; NSAID = non-steroidal anti-inflammatory drug; PPI = proton pump inhibitor; EAC = esophageal adenocarcinoma. Statistically significant differences are printed in bold typing (*p*-value < 0.05).

**Table 2:** Distribution of HGD/EAC between sexes.

Neoplastic Progression	Male (*n* = 639)	Female (*n* = 229)
HGD	37 (71%)	4 (44%)
EAC	15 (29%)	5 (56%)
Total	52 (100%)	9 (100%)

EAC = esophageal adenocarcinoma; HGD = high-grade dysplasia.

**Table 3:** Sex difference in the probability of and time to neoplastic progression.

Probability of Neoplastic Progression (HR; 95% CI) *		
	HGD/EAC	HGD
Female	Ref.	Ref.
Male	2.26 (1.11; 4.62)	3.76 (1.33; 10.6)
Time to Neoplastic Progression (AR; 95% CI) *		
	HGD/EAC	HGD
Female	Ref.	Ref.
Male	0.52 (0.29; 0.95)	0.40 (0.19; 0.86)

AR = acceleration rate; EAC = esophageal adenocarcinoma; HGD = high-grade dysplasia; HR = hazard ratio; Ref = reference category. * Adjusted for sex, BE length, age and the presence of esophagitis.

## Data Availability

The data presented in this study are available on request from the corresponding author.

## Supplementary Materials

The following supporting information can be downloaded at: https://www.mdpi.com/article/10.3390/cancers14133240/s1, Table S1: Sensitivity analysis of sex difference in probability of and time to neoplastic progression.
