# Supplementary material for: Sex Differences in Neoplastic Progression in Barrett’s Esophagus: A Multicenter Prospective Cohort Study
Source: Cancers (Basel). 2022 Jul 1;14(13):3240. doi: 10.3390/cancers14133240 (PMC9264818; doi:10.3390/cancers14133240)
Supplement: Supplementary file 1 [file cancers-14-03240-s001.zip › cancers-1722215-supplementary.pdf]

Supplementary Materials

Sex Differences in Neoplastic Progression in Barrett's Esophagus: A Multicenter Prospective Cohort Study

Carlijn A. M. Roumans, Pauline A. Zellenrath, Ewout W. Steyerberg, Iris Lansdorp-Vogelaar, Michael Doukas, Katharina Biermann, Joyce Alderliesten, Gert van Ingen, Wouter B. Nagengast, Arend Karrenbeld, Frank ter Borg, Mariska Hage, Pieter C. J. ter Borg, Michael A. den Bakker, Alaa Alkhalaf, Frank C. P. Moll, Lieke Brouwer-Hol, Joop van Baarlen, Rutger Quispel, Arjan van Tilburg, Jordy P. W. Burger, Antonie J. P. van Tilburg, Ariadne H. A. G. Ooms, Thjon J. Tang, Mariëlle J. L. Romberg-Camps, Danny Goudkade, Marco J. Bruno, Dimitris Rizopoulos and Manon C. W. Spaander

Table S1. Sensitivity analysis of sex difference in probability of and time to neoplastic progression.

	**Probability of Neoplastic Progression (HR; 95% CI)** †	
	HGD/EAC	EAC
Female	Ref.	Ref.
Male	1.88 (0.91; 3.88)	3.18 (1.12; 9.01)
	**Time to Neoplastic Progression (AR; 95% CI)** †	
	HGD/EAC	EAC
Female	Ref.	Ref.
Male	0.52 (0.34; 1.10)	0.46 (0.22; 0.96)

AR = acceleration rate, EAC = esophageal adenocarcinoma, HGD = high-grade dysplasia, HR = hazard ratio, LGD = low-grade dysplasia. Ref = reference category. † Adjusted for sex, BE length, age and the presence of baseline LGD.
